# Inferior-to-Superior Dissection for Recurrent Laryngeal Nerve Identification in Redo Thyroid Surgery: Enhanced Safety and Reduced Injuries

**DOI:** 10.3390/jcm13237364

**Published:** 2024-12-03

**Authors:** Serdar Gumus, Cemil Yuksel, Huseyin Pulat, Cuneyt Akyuz, Mehmet Onur Gul

**Affiliations:** 1 Department of Surgical Oncology, Mersin City Education and Research Hospital, 33010 Mersin, Turkey; cemil8537@hotmail.com (C.Y.); drhpulat@hotmail.com (H.P.); cuneyt_akyuz@yahoo.com (C.A.); 2 Department of Surgical Oncology, Gaziantep City Education and Research Hospital, 27470 Gaziantep, Turkey; mehmetonurgul@hotmail.com

**Keywords:** hypocalcemia, recurrent laryngeal nerve injury, thyroidectomy

## Abstract

**Background:** Hoarseness due to recurrent laryngeal nerve (RLN) injury is the most feared complication of thyroid surgery. Scars and anatomical changes caused by previous surgeries make finding the RLN during redo thyroid surgeries difficult. We aimed to analyze the results of the inferior-to-superior dissection technique that we applied to find the RLN in redo surgeries. **Methods:** We analyzed the results of 40 consecutive redo thyroidectomy cases in which the inferior-to-superior nerve dissection technique was used to identify the RLN. We compared this cohort with primary thyroidectomies using a lateral-to-medial approach to determine the reliability of this technique. **Results:** Most patients were women (80%), and the mean age was 48.1 years. The ASA score was mostly 2. In total, 25% of the patients had a preoperative diagnosis of malignancy. A total of 8 of the patients underwent unilateral surgery and 32 underwent bilateral surgeries. Two patients had previous recurrent laryngeal nerve paralysis (RLNP), but one of them underwent contralateral surgery. Permanent recurrent laryngeal nerve paralysis developed in only 2 of 71 RLNs at risk (2.8%). Complications classified as Clavien-Dindo 3 and above were observed in 12.5% of our patients during the early postoperative period. The transient hypocalcemia rate was 7.5%, and the permanent hypocalcemia rate was 5%. A 2.8% unilateral RLPN rate was detected, but bilateral RLNP was not observed. All of the complications were not observed to be statistically different among those who underwent primary thyroidectomy. **Conclusions:** The inferior-to-superior nerve dissection approach is a beneficial technique in redo thyroidectomy for preserving RLNP. Surgeons should keep this technique in mind to prevent hoarseness.

## 1. Introduction

The recurrent laryngeal nerve (RLN) is the most essential structure regarding postoperative complications of thyroid surgery. Impairment in voice function after recurrent laryngeal nerve paralysis (RLNP) due to surgery can significantly affect quality of life. The actual incidence of RLNP is unknown. In the reported series, rates of RLNP after primary thyroidectomies are approximately less than 5% [1].

Recurrent thyroidectomies are complicated surgeries due to anatomical changes, fibrous tissue, and scarring from previous operations [2,3]. In cases where redo surgery is required, other factors that make surgery difficult are the recurrence of a goiter mass during the scar formation process after surgery, abnormalities in the anatomy of the nerve due to wound contractions, and changes in the cervical course [4]. RLN visualization is not easy while dissecting scar tissue during redo surgery. Therefore, the risk of RLN injury is the primary concern of re-operative thyroid surgery. Such procedures are technically complex and involve a higher risk of complications than primary procedures [4].

Intra-operative nerve monitoring (IONM) devices are broadly recognized tools for visual identification of the RLN during thyroidectomy. In redo thyroidectomies, IONM is beneficial for functionally detecting the RLN and verifying its integrity, such as during primary surgery. Nevertheless, intraoperative visual identification of the RLN is still the gold standard for preventing RLNP. Visual identification may be performed with correct exposure to prevent nerve injury and preserve nerve integrity. Some approaches have been described to find the RLN, including the following: (1) superior-to-inferior, (2) inferior-to-superior, (3) lateral-to-medial, and (4) medial-to-lateral dissection. In the inferior approach, the RLN is located at the thoracic inlet and dissected upwards for safer identification. In this way, the nerve can be found at the level of the thoracic inlet, which is often an untouched virgin area [5,6].

This study presents the results of 40 consecutive cases in which the inferior dissection technique was used in redo thyroidectomy. We compared the inferior-to-superior technique (in redo thyroidectomy) with the lateral-to-medial approach that we used in primary thyroidectomy and investigated whether the complication and nerve injury rates were worse than those in primary surgery.

## 2. Materials and Methods

This is a retrospective study where the institutional registry at the Surgical Oncology Department, Mersin City Education and Research Hospital, was thoroughly revised for recurrent thyroid surgery cases that were treated at the hospital from September 2021 to April 2024.

### 2.1. Operative Technique

All operations were performed by two surgeons (SG and CY). In redo surgeries, a new operation was performed over the old incision scar. Dissection was initiated lateral to the midline, dividing the infrahyoid muscles in the middle, thus avoiding the fibrous tissue surrounding the thyroid remnant that had been partially removed in the previous operation. If the lobe to be resected had not been disturbed during the prior operation, dissection was carried through the infrahyoid muscles to avoid fibrosis around the anterior face of the trachea. In the first step, the middle thyroid vein was ligated if present. The second step was to find and ligate the superior thyroid veins. In the second step, special attention was always paid to the superior parathyroid gland and the external branch of the superior laryngeal nerve. Then, the RLN was clearly identified in the lower part of the neck and followed along its cervical course. The RLN dissection technique is described below. The valsalva maneuver was routinely performed to detect hemorrhages after thyroidectomy was completed. In all cases, the incision was closed after hemovac drain inserts.

### 2.2. Detection of the RLN with Inferior-to-Superior Approach

**Figure 1:**
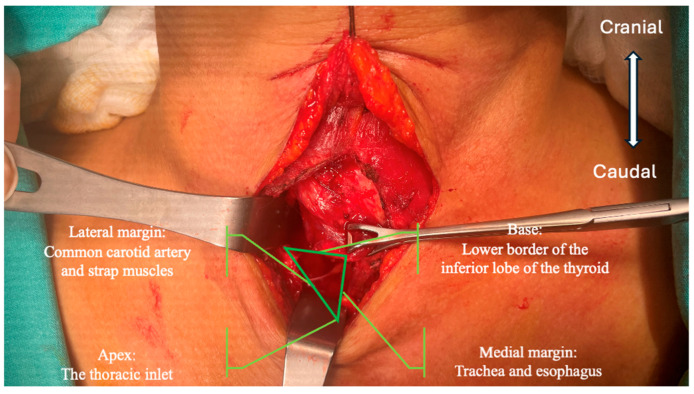
Lore’s triangle.

**Figure 2:**
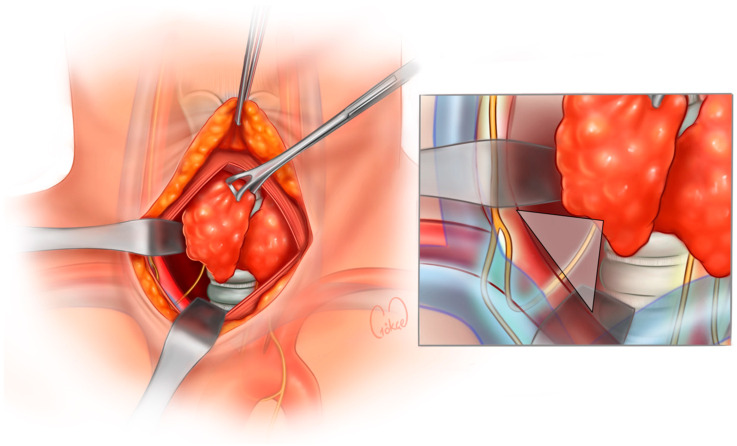
Demonstration of the RLN on Lore’s triangle.

In this approach, after the lateral retraction of the strap muscles, the RLN is detected from its entrance in the ‘virgin’ thoracic inlet region, which is devoid of scar tissue. This area is also called the RLN triangle (Figure 1 and Figure 2). The base of this area is on the cranial, and the top is on the caudal, forming an inverted triangle. The thoracic inlet forms the top of the inverted triangle. The common carotid artery and strap muscles form the lateral edge. The medial edge consists of the trachea and esophagus. If present, the lower border of the lower lobe of the thyroid forms the base of the triangle. The course of the RLN on the right and left is different. While it courses in an area more lateral to the thoracic inlet on the right, it courses in an area closer to the paratracheal area on the left. Extra-laryngeal branching of the RLN within this triangular area is rare and is often found as a single trunk. Therefore, its laryngeal course can be easily visualized.

### 2.3. Identification of Parathyroid Glands

**Figure 3:**
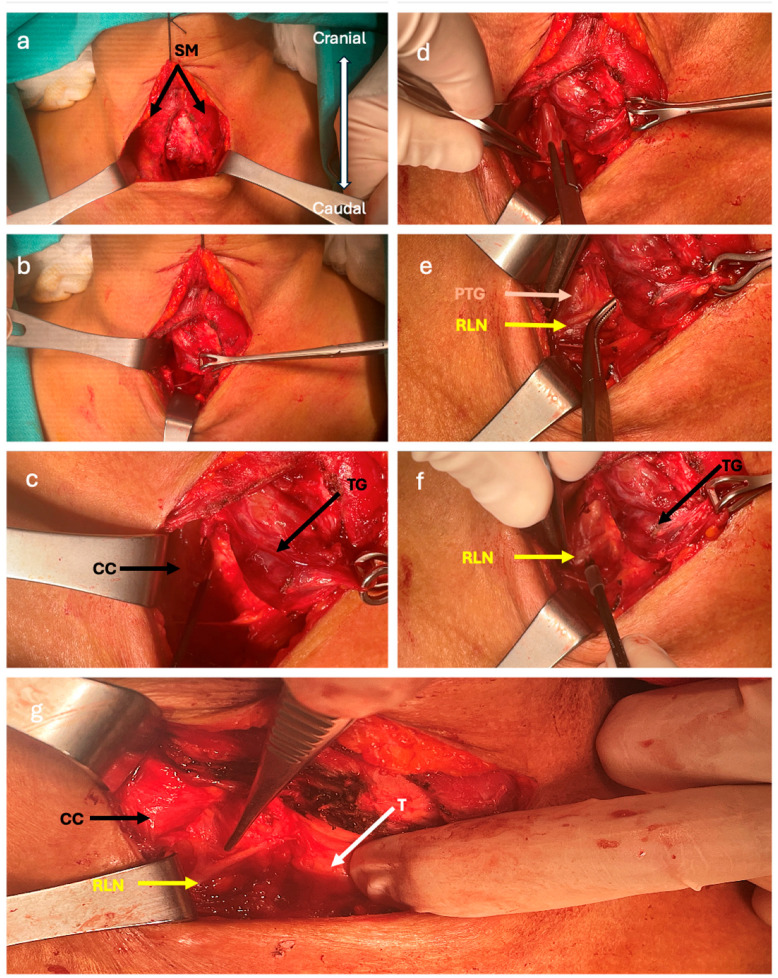
Inferior-to-superior dissection approach steps. The image shows the right-side dissection of a redo thyroidectomy. The RLN was dissected from caudal to cranial in Lore’s triangle. The white arrow indicates the trachea (T), the yellow arrow indicates the RLN, and the beige arrow indicates the lower parathyroid gland (PTG). CC is the common carotid artery, SM is the strap muscles, and TG is the inferior pole of thyroid gland (**a**). Retracted strap muscles. (**b**,**c**) The lower border of the recurrent thyroid is pulled superiorly and medially with Babcock. (**d**,**e**) Lore’s triangle is dissected with a right-angle clamp, and the RLN is dissected from caudal to cranial. (**f**) The nerve is detected with IONM. (**g**) The nerve extends from inferior to superior.

**Figure 4:**
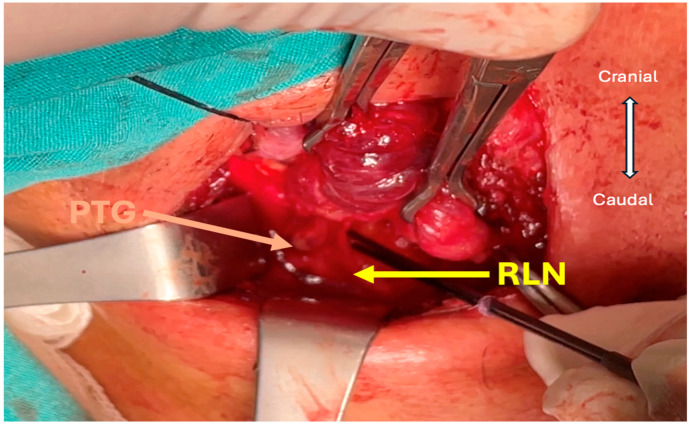
Identification of parathyroid glands. The yellow arrow indicates the RLN, and the beige arrow indicates the lower parathyroid gland (PTG).

The inferior parathyroid vein usually runs from lateral to medial; therefore, when trying to find the RLN, the parathyroid is at risk of vascularization. To avoid this risk, we often push the parathyroid tissue towards the lateral side after detecting the RLN in Lore’s triangle with IONM. Then, we continue the dissection towards the cranial region towards Berry’s ligament (Figure 3e and Figure 4).

### 2.4. Intraoperative Neuromonitoring of the RLN

We used an intermittent intraoperative neuromonitoring (IONM) device to functionally identify the nerve and determine the motor function and functional integrity of the RLN. Intraoperatively, the device produces a sound signal of motor electrophysiological activity, while the EMG signal is recorded as a wave amplitude. The sound signal and the recording of electromyographic amplitude represented the RLN’s proper function. Intermittent IONM was used in each stage of the dissection. First, the Vagus nerve was stimulated with a current of 1–2 milliamperes (mA). Then, nerve stimulation was investigated in a wide area with a current of 1–2 mA before dissecting Lore’s triangle. After Lore’s triangle was dissected, the nerve was found in a narrow area with 0.5–1 mA of current. After the nerve was visually confirmed, the dissection was continued cranially via the nerve monitor.

### 2.5. Clinical Management of the Patients

All patients were consulted by an otolaryngologist for a vocal cord examination using an indirect laryngoscopy before and after the redo surgery. After the operation, the wound in the cervical region was carefully monitored. Local formations such as bleeding, hematoma, and seroma were recorded. The changes that occurred after the surgical intervention were reported, especially vocal cord paralysis and temporary or permanent hypocalcemia. Transient vocal visitation was determined as unilateral vocal cord paresis on laryngoscopic examination and returned to normal within 6–8 weeks after the operation. Permanent vocal cord paralysis was defined as single paralysis on a laryngoscopic examination six months later.

The presence and type of hypocalcemia signs and an evaluation of Chvostek’s sign were recorded. We discharged the patients once they were asymptomatic or their serum calcium level was normal. In consecutive postoperative measurements, biochemical hypocalcemia was defined as a serum calcium level below 2.0 mmol/L. We described postoperative hypocalcemia as a condition where patients needed medication to maintain normocalcaemia at the time of discharge. During the follow-up period, hypocalcemia was considered transient after all medications had been discontinued and normocalcaemia had been maintained for at least two weeks. Hypocalcemia associated with low parathyroid hormone (PTH) levels (mean: 9–55 pg/mL) was considered permanent if it persisted for more than six months after surgery and required treatment to maintain normocalcaemia.

### 2.6. Statistical Analysis

The Statistic Package for Social Science (SPSS 25.0) (IBM Corporation, Armonk, NY, USA) was used to analyze and report continuous variables as the mean with standard deviation (SD) and categorical variables as frequencies and percentages. Analytical methods were used to determine whether the variables were normally distributed. The chi-square test was used to compare groups. A *p*-value< 0.05 was considered significant.

## 3. Results

A total of 308 thyroid surgeries were performed during the study period. Forty of these were redo surgeries. In patients excluding those with redo surgeries (40 cases), 206 bilateral and 62 unilateral primer thyroidectomies were performed.

In the 40 redo surgery cases included in this study, most of the patients were women (80%), and the mean age was 48.1 years. The ASA score was mostly 2. Twenty-five percent of the patients had a preoperative diagnosis of malignancy, and 40% had suspected malign nodules upon fine-needle aspiration cytology. The most commonly performed procedure was total thyroidectomy (Table 1). Eight of the patients had undergone neck surgery twice, two had the procedure three times before our surgery, and the remaining thirty patients had thyroid surgery once.

Of the 40 patients, 8 were operated on unilaterally and 32 were operated on bilaterally. A total of 71 nerves were at risk in 40 patients, 1 of whom underwent bilateral surgery and already had a unilateral nerve injury. One of the eight patients who underwent unilateral surgery had a previous nerve injury on the opposite side. However, since that side was not dissected in our procedure, we did not evaluate it as a nerve at risk. Only 2 of the 71 nerves at risk were found to be damaged. The new RLPN rate was 2.8% in all cohorts, and there was no bilateral RLNP. The total number of nerves at risk in primary thyroidectomies was 474. According to the cases with the RLN at risk, the RLNP rate was 1.28% in primary surgeries (six cases). It was determined that there was no statistical difference between the two groups (*p* = 0.311) (Table 2).

Complications classified as Clavien-Dindo 3 and above were seen in 12.5% of our patients during the early postoperative period. Our early transient hypocalcemia rate was 7.5%. In the entire cohort, one patient developed a wound hematoma and was drained. One patient developed a lung effusion and was drained percutaneously. In one patient who had a lateral neck dissection and had previously received RAI treatment, a wound infection was observed, which was then corrected with antibiotics. Two patients had chylous leakage. One had a left lateral dissection, and the other had only a central lymphatic dissection with total thyroidectomy. They were treated with a medium-chain fat diet until the chylous leakage resolved. Only chylous fistula was statistically significantly more common in repeat thyroid surgeries. This was due to the left neck dissection cases performed in repeat surgeries. Other complications, such as hypocalcemia, were not statistically significant, although they were at lower percentages (Table 3).

## 4. Discussion

This study presents the surgical outcomes of the inferior-to-superior RLN identification approach in redo thyroid surgeries. The outcomes of this approach were investigated through a retrospective review of two surgeons’ cohorts of patients. The rate of permanent vocal cord palsy was 2.8%, while the rates of transient and permanent hypocalcemia were 7.5% and 5%, respectively.

Redo thyroid surgeries have technical challenges that require high-level surgical experience. Adhesions, fibrosis, and scars from previous surgeries may cause changes in the anatomical line of the RLN during redo thyroid surgery [5,6,7]. On the other hand, the location of the parathyroid glands may change and be challenging to find in redo surgery. Different surgical approaches to finding recurrent laryngeal nerves depend on the surgeon’s habit. The superior approach, the lateral approach, the inferior approach, and the medial approach were described. Among previously described dissection techniques, the inferior approach recommended by the redo-surgery iteration is the first. The inferior area is often not dissected in previous surgeries. Therefore, this virgin area usually has less adhesion and scarring. The importance of this technique lies in starting the operation from the most inferior area that has not been dissected in previous surgeries and has less adhesion because no surgeon needs to go down that far during primary thyroidectomy. Recurrences often occur around the Berry ligament, and possible nerve injury may occur during the dissection of that area with a superior or lateral approach. Lore and colleagues described this virgin triangle, known as the RLN triangle, as follows: The area’s medial border is the trachea, the lateral border is the laterally retracting strap muscles and common carotid artery, and the base of the triangle is the inferior pole of the thyroid. The apex of this triangle faces the thoracic inlet, and the base is cranial [6]. The literature includes articles that report the use of inferior dissection techniques in revisional surgeries. However, these articles do not go beyond technical descriptions [8,9,10]. Some non-comparative studies have examined this technique in more detail. In 2021, Lee and colleagues conducted a study that addressed the dissection of the RLN from inferior to superior [11]. According to their study, the nerve can be easily found in the area they defined (lower central triangle), similar to Lore’s triangle. In another study, Miyauchi and colleagues defined the most inferior approach (ima approach) technique by starting the inferior approach even lower [12]. The common goal of these studies is to provide standardized nerve identification and minimize injury rates.

Although an article compared the superior approach with the lateral approach [13], no article compared the inferior dissection technique with other techniques. In the present study, we compared the complication rates of the primary surgery cohort performed during the same period to demonstrate the safety of the inferior-to-superior dissection technique. The only statistical difference between the complications of this technique and the primary surgeries was related to the chylous fistula. This was due to the left neck dissections performed during redo surgeries. Neither nerve injury nor calcium metabolism changes were different.

It will be favorable to examine the complication rates in the literature to understand the success of our cohort. A comprehensive Scandinavian multicenter audit showed that after primary thyroidectomy, the unilateral RLNP rate was 3.9%, and the bilateral was 0.2% [14]. In another retrospective comparative German study, the RLNP rate was near 3.6% in re-operative thyroid surgery. Our study’s RLNP rate was 2.8% in redo surgery, less than the presented primer or redo surgery.

Concerning other surgical complications, hypocalcemia is essential in redo thyroid surgery as well. Kim et al. presented the results of 65 patients who underwent redo operations, with the transient hypocalcemia rate being 26.2% and the permanent hypocalcemia rate being 4.6% in their series [15]. In our series, the transient rate was less (7.5%), and the permanent rate was similar (5%) in redo surgery. Although our redo surgery percentages seem to be slightly higher than those for primary surgeries, they are quite acceptable for redo surgeries.

Different IONM devices have been developed to minimize RLN damage. However, their effectiveness is still controversial, and there is a consensus that the best way to preserve the RLN is to visualize it. Although a large meta-analysis found that IONM reduces RLNP, it was emphasized that further studies are needed due to the unreliability of the data. In this German study, intraoperative nerve monitoring was used in 91 redo thyroid operations, whereas 159 were performed with direct nerve visualization alone [16]. The authors noted that intraoperative nerve monitoring does not reduce the rate of RLNP. A comprehensive meta-analysis by Henry et al. confirmed that IONM did not reduce RLNP with statistical significance [17]. We believe that the use of IONM tools helps find the nerves. However, the literature does not always support that their use helps prevent RLNP, so it is challenging to say whether IONM prevents RLNP rates in thyroidectomy. Therefore, an ideal nerve visualization and dissection strategy is still needed. We routinely use IONM in our technique, and we believe that its primary benefit is that it first guides the identification of the nerve in the Lore triangle during redo surgery. IONM alone will never be able to prevent RLNP in surgeries performed without appropriate techniques. Therefore, we believe surgeons should know the nerve-finding methods separately for each case.

In evaluating special points, the non-recurrent laryngeal nerve is a risk for this dissection technique. Therefore, this risk can be eliminated by correctly using the IONM technique. The entrance of the RLN to both thyroid regions is different; while the RLN is sought more lateral to the thoracic inlet on the right side, it is sought in an area closer to the paratracheal area on the left. Extra laryngeal branching is rare in this region, and the RLN is found as a single trunk; therefore, its laryngeal course can be visualized more easily. However, it has been reported that dissection in the long segment may increase the risk of nerve injury [18]. Typically, the inferior parathyroid vessel runs in a lateral to medial direction; therefore, when we try to find the RLN, there is a risk of parathyroid re-vascularization. On the other hand, the inferior approach may have some limitations, such as large, substernal goiters and the presence of a non-RLN.

The present study has important sides. First, this technique is a known and described approach used by experienced thyroid surgeons. However, the surgical literature has not yet described and demonstrated its effectiveness enough. Second, this study reports on the homogenous-compared cohort in the literature for whom the inferior approach to identifying the RLN was used.

## 5. Limitations

This study has a few limitations. First, it is retrospective. Second, this study represents two surgeons’ experiences, possibly limiting the generalizability of the findings. Most patients in this study had malignant nodules or suspected malignancy and no giant or large substernal goiters. This technique may have limitations in these cases, which is a limitation of our study. Finally, it is not known exactly whether this method has been used in other published series on revision thyroid surgery and/or in what proportion of cases it has been used; therefore, our limitation is that we cannot expand our discussion with prospective and retrospective studies in the literature.

## 6. Conclusions

The inferior approach is a surgical technique that can be used in redo thyroid surgeries to find the RLN. Its complication rates are not higher than those of primary thyroidectomy.

## Figures and Tables

**Table 1:** Demographic and clinical features.

Variables	Values (*n*)	Rate (%)
Mean age (years)	48.1	
Female gender	32	80%
ASA * Score		
ASA1	4	10%
ASA2	24	60%
ASA3	12	30%
Indication for redo surgery		
Benign	10	25%
Suspected malignant nodule	16	40%
Malignant	10	25%
Recurrence of Grave’s disease and Hashimoto thyroiditis	4	10%
Type of surgery		
Unilateral lobectomy	8	20%
Total thyroidectomy	16	40%
Total thyroidectomy + sentral neck dissections	12	30%
Total thyroidectomy + lateral neck dissections	4	10%

* ASA, American Society of Anesthesiologists.

**Table 2:** Clinical features of nerves.

	Side	Redo			Primary			*p*
		Nerves at Risk	Previous RLNP	New RLNP	Nerves at Risk	Previous RLNP	New RLNP	
Unilateral	Left RLN	2	1 *	0	28	0	0	
	Right RLN	6	0	1	34	0	0	
Bilateral	Left RLN	32	0	1	206	0	4	
	Right RLN	31	1	0	206	0	2	
Totaly		71	2	2 (2.8%)	474	0	6 (1.3%)	0.311

* One patient has previous contralateral RLNP and is therefore deemed not at risk.

**Table 3:** Early and late postoperative complications.

	Clavien-Dindo Score	Redo Surgery *n* = 40, (%)	Primer Surgery *n* = 268, (%)	*p*
Temporary hypocalcemia	2	3 (7.5%)	17 (6.3%)	0.782
Permanent hypocalcemia	-	2 (5%)	6 (2.2%)	0.306
Hematoma	3b	1 (2.5%)	4 (1.5%)	0.638
Voice changes	1	3 (7.5%)	9 (3.4%)	0.602
Chest effusion	4b	1 (2.5%)	2 (0.7%)	0.292
Chyle leak	3a–3b	2 (5%)	1 (0.4%)	0.005
Wound infection	3a	1 (2.5%)	4 (1.5%)	0.638
Severe neck edema	1	1 (2.5%)	0 (0%)	Na

## Data Availability

All data will be shared if requested.
